# Tracking the evolution of tuberculosis research priorities in Africa: a longitudinal bibliometric analysis from 2003–2023

**DOI:** 10.3389/frma.2026.1931249

**Published:** 2026-09-09

**Authors:** Olusesan Adeyemi Adelabu, Peter Ayodeji Idowu, Jolly Musoke

**Affiliations:** 1 Department of Medical Microbiology, School of Pathology, Faculty of Health Sciences, University of the Free State, Bloemfontein, South Africa; 2 Section Veterinary Public Health, Department of Paraclinical Science, Faculty of Veterinary Medicine, University of Pretoria, Onderstepoort, South Africa; 3 National Health Laboratory Service, Department of Medical Microbiology, School of Pathology, University of the Free State, Bloemfontein, South Africa

**Keywords:** Africa, bibliometrics, biblioshiny, mycobacterium tuberculosis, tuberculosis

## Abstract

**Background:**

*Mycobacterium tuberculosis* is the primary causative agent of pulmonary tuberculosis and remains a major global public health concern, particularly in low- and lower-middle-income countries. Tuberculosis continues to be one of the leading causes of death from infectious diseases and is a significant contributor to mortality among both HIV-positive and HIV-negative populations in Africa. Despite extensive epidemiological research on tuberculosis, bibliometric analyses focusing on African research output remain limited. This study therefore examined trends, patterns, and research gaps in tuberculosis-related publications across African countries.

**Methods:**

A bibliometric analysis was conducted using the Scopus database for publications between January 2003 and December 2023. The study selection process followed PRISMA guidelines. A total of 8,797 records were identified, of which 8,181 met the inclusion criteria after screening and eligibility assessment. Data were analyzed using RStudio (Bibliometrix package and Biblioshiny interface) and Microsoft Excel 2023. Analyses included authorship patterns, institutional and country contributions, publication trends, citation analysis, collaboration networks, leading journals, funding organizations, and thematic mapping.

**Results:**

Tuberculosis research output in Africa increased consistently over the study period. Articles accounted for most publications (75%), followed by reviews (10%) and conference papers (2%). South Africa was the most productive and most cited country, while the University of Cape Town demonstrated the strongest institutional collaboration network. Highly cited contributions included studies by Hoosen Coovadia et al. (2009) and Abdelkrim Boutayeb (2006). *PLOS ONE* published the highest number of articles, followed by the *South African Medical Journal* and the *International Journal of Tuberculosis and Lung Disease*. Collaboration networks showed strong intra-African and international linkages. Key research themes included HIV/TB co-infection, drug resistance, epidemiology, and public health interventions.

**Conclusion:**

Tuberculosis research in Africa has increased substantially over the past two decades, with growing emphasis on epidemiology, drug resistance, and HIV co-infection. South Africa remains the leading contributor, likely reflecting stronger research infrastructure and funding capacity. However, gaps persist in regionally balanced research output and collaboration. Strengthening intra-African and global partnerships, as well as supporting underrepresented regions, will be critical for advancing tuberculosis research and control efforts across the continent.

**Systematic review registration:**

https://doi.org/10.5281/zenodo.21672964

## Introduction

Pulmonary tuberculosis (TB) is an infectious disease that primarily affects the lungs via an infectious agent known to be *Mycobacterium tuberculosis*; it is transmitted through the air from the cough of an infected individual. Globally, TB is still considered one of the oldest known diseases and poses a serious threat to public health (Ong et al., 2020). The leading cause of death from infectious diseases, with 1.6 million cases and an incidence of 10 million new cases worldwide (Garrido-Cardenas et al., 2020), has been implicated in various geographical regions of the world (WHO, 2023). According to the World Health Organization Global Tuberculosis Report 2024, Africa remains one of the regions most affected by tuberculosis, accounting for a substantial proportion of global TB incidence and mortality.

In addition, a quarter of all new tuberculosis cases in 2022 occurred in Africa, where 2.5 million cases were reported. Globally, there were an estimated 1.13 million deaths among HIV-negative people in 2022. The disease has claimed the lives of 424,000 people on the African continent (1.267 million worldwide). Over 33% of tuberculosis-related deaths occur on the African continent. However, the heavy burden of HIV on the continent is reflected in the 20% of new TB cases reported among individuals with HIV and AIDS (WHO, 2023). The high mortality, morbidity, and incidence rates of Drug-resistant TB disease occur when Mycobacterium tuberculosis becomes resistant to first-line anti-tuberculosis drugs, including multidrug-resistant (MDR-TB) and extensively drug-resistant TB (XDR-TB) (Silva et al., 2021).

The *Mycobacterium* genus has more than 140 species (Ledesma et al., 2022) and is divided into three major groups: the *Mycobacterium tuberculosis complex* (MTBC), *M. leprae*, and non-tuberculous Mycobacteria. The greatest risk factors for developing tuberculosis disease include close contact with infectious TB cases, HIV infection, diabetes mellitus, malnutrition, and immunosuppression (Ledesma et al., 2022; Kanabalan et al., 2021). Overcrowded living conditions increase risk of infection but do not independently increase progression to active disease (Igwaran and Edoamodu, 2021; Obeagu and Obeagu, 2023; Gelaw et al., 2021). Additionally, efforts have been made globally to mitigate the effects of TB, but the infection continues to constitute a significant concern for public health in many geographical regions worldwide owing to the development of multidrug-resistant *M. tuberculosis* (MDR-MTB), which occurs when MTB strains become resistant to antimicrobials or antibiotics, especially rifampicin and isoniazid, which are primarily designed for the treatment of this disease. The increase in the incidence of MDR-TB has become one of the factors responsible for the resurgence of the TB epidemic globally (Xi et al., 2022; Harichander et al., 2022; Naidu et al., 2020).

Bibliometrics has become a well-known and rigorous tool for exploring and analyzing large volumes of scientific metadata, and it is effective in unpacking evolutionary distinctions in scientific research and related fields while shedding light on the emerging trends and topics in such fields. Researchers employ bibliometric analysis for several purposes, including identifying new trends in the performance of articles and journals, patterns of collaboration, and the components of research, as well as investigating the intellectual framework of a particular field in the literature (Donthu et al., 2021; Tomaszewski, 2023). Additionally, in-depth data analysis, visualization, and mapping have led to the emergence of bibliometric research from laborious manual item counts (Lim and Kumar, 2024). The resulting information derived from the maps and visualization can be utilized to determine potential future research orientations and collaborative prospects among researchers.

Although previous studies have examined tuberculosis research in Africa, most have focused on relatively short time periods or provided cross-sectional snapshots of publication output, authorship, and citation patterns. Consequently, there remains limited understanding of how research priorities, collaboration networks, funding patterns, and thematic structures have evolved over 20 years in response to major scientific, technological, and public health developments. A comprehensive longitudinal assessment is important due to substantial changes over the past two decades in African tuberculosis research landscape, including advances in molecular diagnostics, the increasing burden of multidrug-resistant tuberculosis, evolving HIV/TB co-infection research, expanded international collaborations, and disruptions associated with the COVID-19 pandemic. Understanding these temporal changes provides insights that cannot be obtained from cross-sectional bibliometric analyses alone and helps identify emerging research priorities, persistent knowledge gaps, and opportunities for future investment.

Therefore, this bibliometric study aimed to analyze the evolution of tuberculosis research in Africa from 2003–2023 with a focus on three core research questions:

1. How have tuberculosis research outputs and thematic priorities changed over time in Africa?
2. How have collaboration networks among African countries, institutions, and authors evolved during the study period?
3. What are the major funding sources and how have funding patterns influenced research productivity?

By adopting a 20-year longitudinal perspective, this study moves beyond describing publication trends to examine how the African tuberculosis research ecosystem has changed over time. Specifically, it integrates temporal thematic evolution, collaboration network dynamics, institutional productivity, funding patterns, and fractionalized citation impact to provide a more comprehensive understanding of the structural evolution of tuberculosis research in Africa. These findings offer evidence that can support researchers, funding agencies, and policymakers in identifying research strengths, addressing persistent gaps, and prioritizing future investments in tuberculosis research and control across the continent.

By adopting a 20-year longitudinal perspective, this study moves beyond the cross-sectional bibliometric analyses previously reported for tuberculosis research in Africa. It integrates temporal thematic evolution, collaboration network dynamics, institutional productivity, funding patterns, and fractionalized citation impact to provide a more comprehensive understanding of how the African tuberculosis research ecosystem has evolved. These findings provide evidence to support researchers, funding agencies, and policymakers in identifying research strengths, persistent gaps, and future investment priorities.

## Study design

This study employed a longitudinal bibliometric analysis to evaluate trends, collaboration networks, thematic evolution, and funding patterns in tuberculosis research in Africa between 2003 and 2023. Bibliometric methods are widely used to analyze scientific metadata and to identify structural and temporal patterns in research fields. The period 2003–2023 was selected as it captures major milestones in tuberculosis control and research in Africa, including:

- expansion of DOTS-Plus treatment programmes,
- increased HIV/TB co-infection research,
- introduction of GeneXpert diagnostic technology around 2010,
- growth of MDR-TB surveillance programmes,
- and disruptions to TB services during the COVID-19 pandemic.

Analyzing this 20-year period therefore allows evaluation of long-term changes in research priorities across key technological and policy transitions.

### Database selection

Scientific publications were retrieved from the Scopus database (Elsevier), accessed in August 2024. Scopus was selected as it provides extensive coverage of peer-reviewed journals, conference proceedings, and citation metadata suitable for bibliometric analysis, as well as analytical tools for examining author affiliations, institutional collaboration, and funding sources. In addition, Scopus allows forward and backward citation tracking, enabling longitudinal assessment of research influence across time (Burnham, 2006). Scopus was selected because it provides broad multidisciplinary coverage and detailed citation metadata suitable for bibliometric analysis. Comparative studies have shown that Scopus indexes a wider range of journals than Web of Science, although no single database provides complete coverage of the scientific literature (Burnham, 2006; Singh et al., 2021). Nevertheless, reliance on a single bibliographic database may introduce database-specific bias as journal coverage, indexing policies, and citation records differ across databases. Consequently, some regional journals, especially those not indexed in Scopus, may not have been captured, which could influence estimates of publication productivity, citation patterns, and the representation of tuberculosis research conducted in Africa. Despite this limitation, Scopus was considered appropriate based on its extensive multidisciplinary coverage, standardized bibliographic metadata, and compatibility with Bibliometrix for comprehensive bibliometric analyses.

### Search strategy

A comprehensive search strategy was developed using multiple keyword variants to maximize the retrieval of tuberculosis-related publications. The search was performed in the Title, Abstract, and Keywords (TITLE-ABS-KEY) fields using the following query:

TITLE-ABS-KEY (“tuberculosis” OR “TB” OR “MDR-TB” OR “XDR-TB” OR “multidrug resistant tuberculosis” OR “extensively drug-resistant tuberculosis” OR “Mycobacterium tuberculosis” OR “silicotuberculosis”) AND PUBYEAR > 2002 AND PUBYEAR < 2024.

The search incorporated commonly used disease names, abbreviations, and spelling variants to improve sensitivity. Controlled vocabulary terms (MeSH) were not used as Scopus database primarily indexes records using author-provided keywords, titles, and abstracts rather than controlled indexing terms. Following retrieval, records were filtered to include publications with at least one author affiliated with an African institution, thereby capturing research contributions from across the continent without the need to include individual African country names in the search query. No restriction was placed on document type during the initial search to maximize the retrieval of relevant publications. Only English-language publications were included to ensure consistency in keyword extraction, thematic mapping, and bibliometric analyses. Although this language restriction may have excluded some relevant studies published in other languages, English remains the pre-dominant language of scientific publication indexed in Scopus and facilitates reproducibility and comparability of bibliometric analyses.

### Study selection (PRISMA framework)

**Figure 1:**
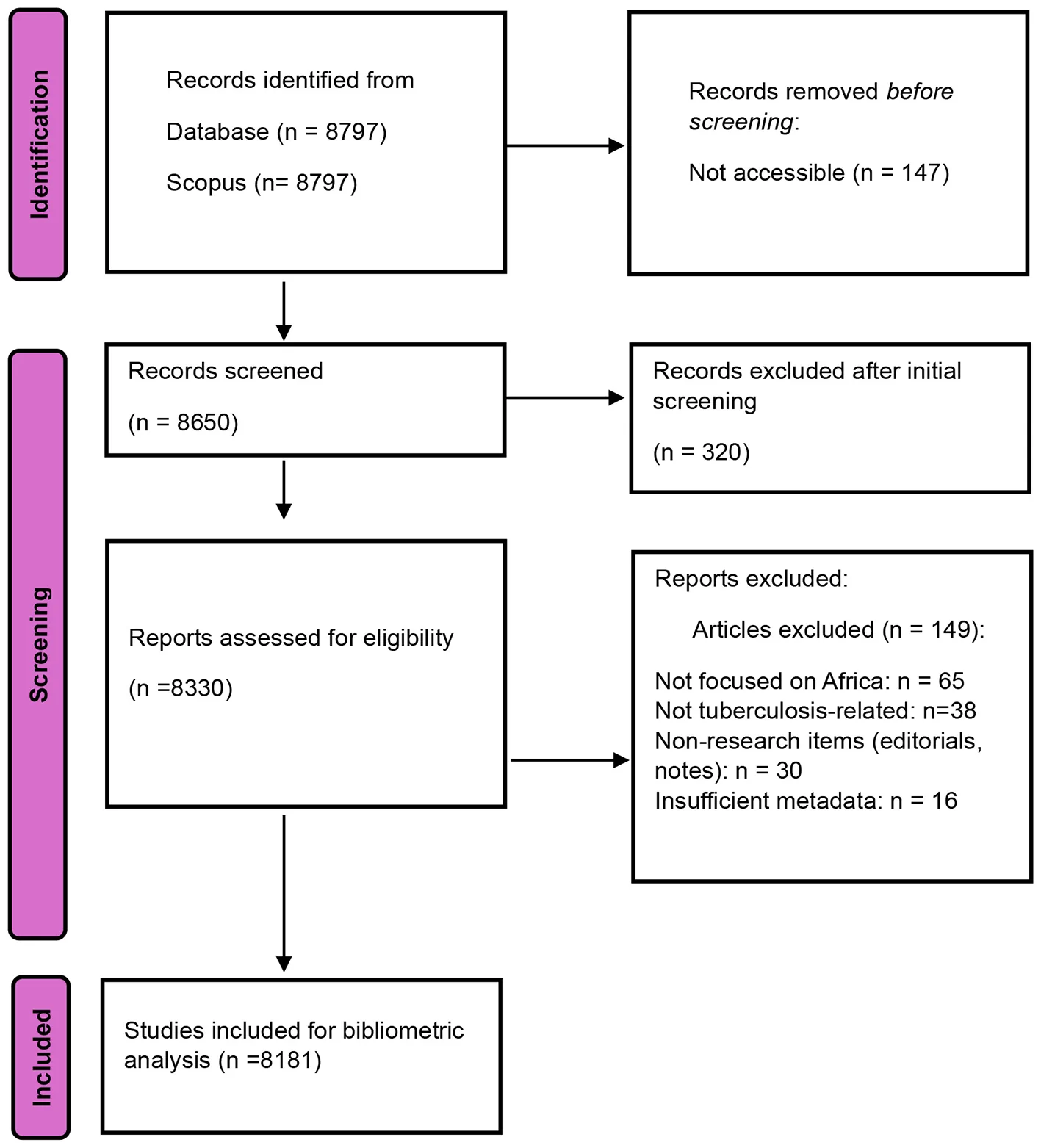
PRISMA flow diagram of study selection for bibliometric analysis of tuberculosis research in Africa (2003–2023).

The study selection process followed the Preferred Reporting Items for Systematic Reviews and Meta-Analyses (PRISMA) guidelines. A total of 8,797 records were initially retrieved from the Scopus database. After removal of duplicate records (*n* = 147), 8,650 records remained for title and abstract screening. During the screening stage, 320 records were excluded due to irrelevance to tuberculosis research or lack of focus on the African context. A total of 8,330 records were subsequently assessed for eligibility. Of these, 149 records were excluded for not meeting the inclusion criteria, including studies not focused on Africa (*n* = 65), non-tuberculosis-related studies (*n* = 38), non-research document types such as editorials and notes (*n* = 30), and records with insufficient bibliometric information (*n* = 16). Ultimately, 8,181 publications were included in the final bibliometric analysis (Figure 1).

### Data extraction and analysis

Bibliographic records were exported in BibTeX format and analyzed using Bibliometrix package in R (v4.3.2) and Biblioshiny interface, Microsoft Excel 2023 for descriptive statistics. The analysis included annual publication and citation trends, leading authors, institutions, and countries, funding sources, collaboration network analysis, keyword co-occurrence and thematic mapping.

### Bibliometric indicators

Several specialized bibliometric indicators were calculated. Fractionalized Citation Count were calculated to account for multi-author publications. For each article:

$$\begin{array}{l} Fractional\;citation\;credit\;for\;author\;i=\frac{Total\;citations\;of\;article}{\text{Number of authors}} \end{array}$$

This approach assigns proportional credit to each co-author and avoids overestimation of productivity in large collaborations.

Author Keywords (DE) are keywords provided by the authors in the original publication. Keywords Plus (ID) are algorithmically generated keywords derived from the titles of cited references. DE keywords reflect author-defined research topics, while ID keywords capture broader thematic associations. Collaboration networks were analyzed using the Walktrap algorithm, which detects communities in a network by simulating short random walks between nodes. Nodes frequently visited together are grouped into clusters, representing closely collaborating authors or institutions. Cluster size, density, and centrality were calculated using standard network metrics within the Bibliometrix package.

### Data screening and quality control

To ensure the accuracy and relevance of the dataset, a multi-stage data quality control procedure was implemented. Records retrieved from Scopus were filtered by year, country affiliation, and keyword search strategy. Random samples of retrieved articles were manually reviewed to confirm that the study topic was tuberculosis. Articles retrieved due to ambiguous abbreviations (e.g., “TB” referring to “terabyte” or “tumor burden”) were excluded. Author affiliations were manually checked for a subset of records to verify country classification. Scopus occasionally misclassifies affiliations or funding agencies; where inconsistencies were detected, records were corrected or excluded. Funding agency names were standardized to merge spelling variations and duplicate funders. If irrelevant records were detected during screening, the search strategy was refined and re-run to improve dataset precision. These procedures ensured that only publications relevant to tuberculosis research conducted in Africa were included.

## Results

From the Scopus web interface, a total of 190,080 scientific documents were found, and after the documents were filtered based on the eligibility indices, 8,181 papers were finally used for the bibliometric study (Table 1). An average of 13.52 citations per document with an annual growth rate of 9.46% were found among the scientific document analyses in this study. Interestingly, articles (*n* = 6,183: 75%) were the most frequently published scientific documents, followed by review papers (*n* = 849:10%), conference papers (*n* = 181:2%). The curated scientific documents also included 21,875 authors, 782 single-authored documents, and 603 authors of single-authored documents, with 11,394 and 25,657 author keywords (DE) and keywords plus (ID), respectively. The overall details of the publications that were curated from the Scopus web interface on tuberculosis between 2003 and 2023 are listed in Table 1.

**Table 1:** General information on curated scientific documents on tuberculosis.

Description	Results
Timespan	2003:2023
Sources (journals, books, etc.)	2,112
Documents	8,181
Average citations per documents	13.52
Annual growth rate %	9.46
Document average age	8.06
International co authorships %	7.053
Article	6,183
Review	849
Conference paper	181
Editorial	171
Book chapter	146
Single-authored documents	782
Authors of single-authored documents	603
Co-Authors per documents	4.65
AUTHORS	21,875
Author's keywords (DE)	11,394
Keywords Plus (ID)	25,657

### Annual trend in scientific production

**Figure 2:**
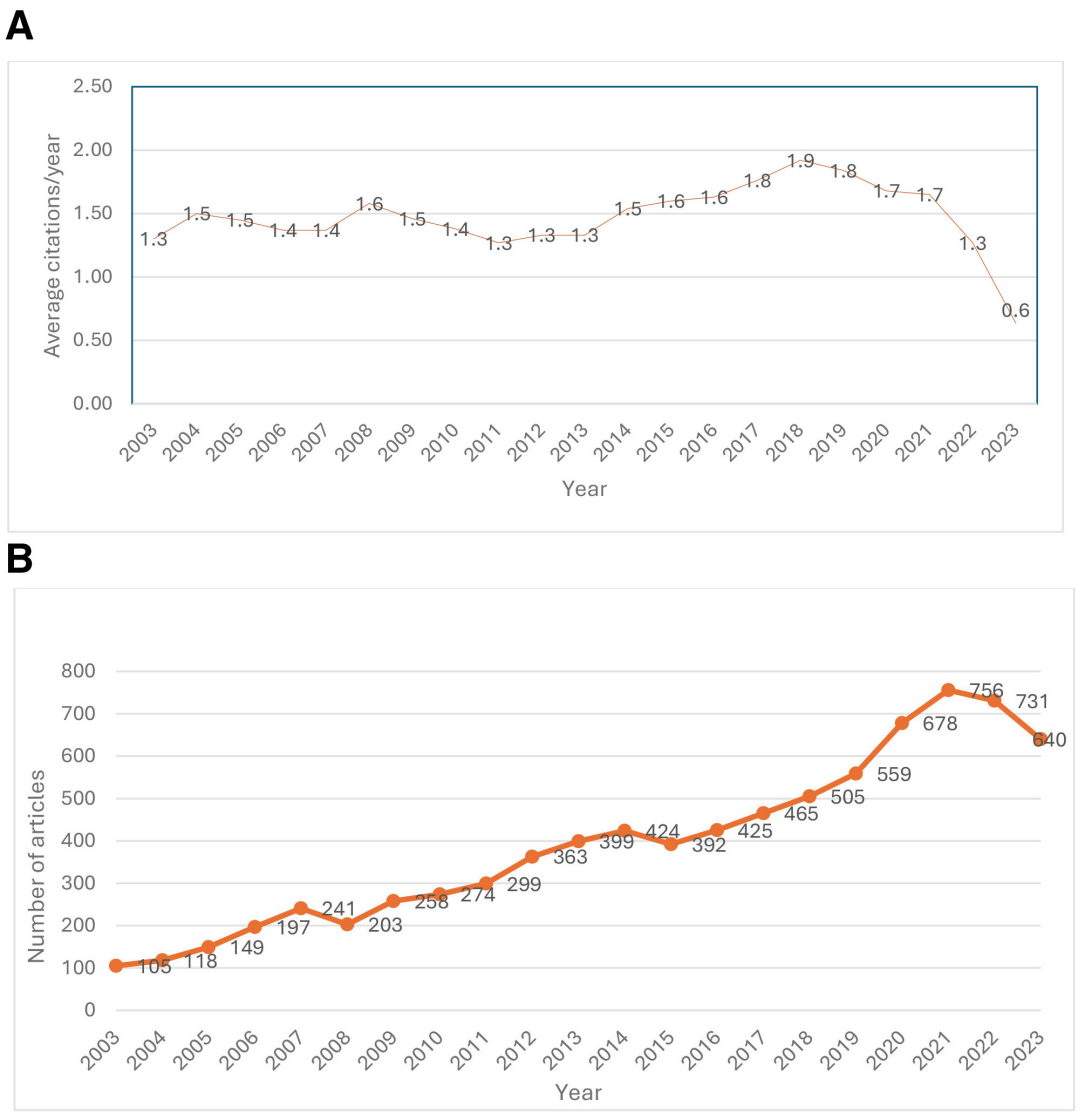
**(A)** Annual publication output on tuberculosis research in Africa between 2003 and 2023. **(B)** Temporal trend in average citations per publication during the study period.

Figure 2A illustrates the annual publication output on tuberculosis research in Africa between 2003 and 2023, showing a consistent upward trend with a slight decline in 2022–2023. Figure 2B presents the temporal trend in average citations per publication, demonstrating that citation impact increased over time despite year-to-year fluctuations. Between 2003 and 2023, there is convincing evidence of a continuous upwards trend in the number of publications in research on tuberculosis in Africa, with the lowest (*n* = 105) number of publications being in the year 2003 and the highest (*n* = 756) number of publications being in the year 2021, although a momentous decline is observed in the years 2022 and 2023, with the production of 731 and 640 articles, respectively (Figure 2A). Conversely, the yearly average citation trend has steadily risen but unevenly, with the mean number of citations reaching a maximum of 1.9 in 2018 (Figure 2B). Additionally, the apparent decline in publications in 2022–2023 likely reflects indexing and publication delays rather than a true reduction in research activity (Figure 2B).

Table 2 displays the 20 top-ranked journals on tuberculosis publications together with the total number of papers published from 2003–2023. *Plos One* journal published the greatest number of scientific papers on the topic of interest (301; 3.7%), followed by the *South African Medical Journal* (292; 3.6%) and the *International Journal of Tuberculosis and Lung Disease* (239; 3.0%), during the study period (2003–2023). Based on the JCR^®^ 2022 impact factor, the journals' respective journal impact factors were 3.7, 2.2, and 4.0. Additionally, *Pan African Medical Journal* and *International Journal of Mycobacteriology* have 238 (3.0%) and 120 (1.4%) published papers, respectively, but their impact factors are not available on the JCR^®^ 2022 Impact Factor. Interestingly, the *International Journal of Infectious Diseases* has the highest impact factor (8.4) but published only 50 scientific papers between 2003 and 2023.

**Table 2:** Topmost journals publishing papers on tuberculosis.

Rank	Sources	h_index	Number of articles published on the topic (%), *n* = 8,181	JCR^^®^^ 2022 impact factor
1	*PLoS ONE*	38	301 (3.7%)	3.7
2	*South African Medical Journal*	34	292 (3.6%)	2.2
3	*International Journal of Tuberculosis and Lung Disease*	29	239 (3.0%)	4.0
4	*Pan African Medical Journal*	27	238 (3.0%)	N/A
5	*BMC Infectious Diseases*	26	156 (2%)	3.7
6	*International Journal of Mycobacteriology*	25	120 (1.4%)	N/A
7	*Southern African Journal of HIV Medicine*	23	102 (1.2%)	1.7
8	*BMC Public Health*	23	95 (1.2%)	4.5
9	*African Health Sciences*	20	90 (1.1%)	1.0
10	*BMC Research Notes*	20	82 (1.0)	N/A
11	*South African Family Practice*	18	70 (0.8%)	N/A
12	*Tuberculosis*	17	64 (0.8%)	3.2
13	*Infection And Drug Resistance*	17	59 (0.7%)	3.9
14	*International Journal of Infectious Diseases*	17	50 (0.6%)	8.4
15	*Journal Of Clinical Tuberculosis and Other Mycobacterial Diseases*	16	48 (0.6%)	N/A
16	*Ethiopian Medical Journal*	15	45 (0.5%)	N/A
17	*African Journal of Primary Health Care and Family Medicine*	15	44 (0.5%)	N/A
18	*HIV/Aids-Research and Palliative Care*	14	42 (0.5%)	N/A
19	*East African Medical Journal*	12	41 (0.5%)	N/A
20	*BMJ Open*	12	40 (0.5%)	4.1

N/A, not available.

**Figure 3:**
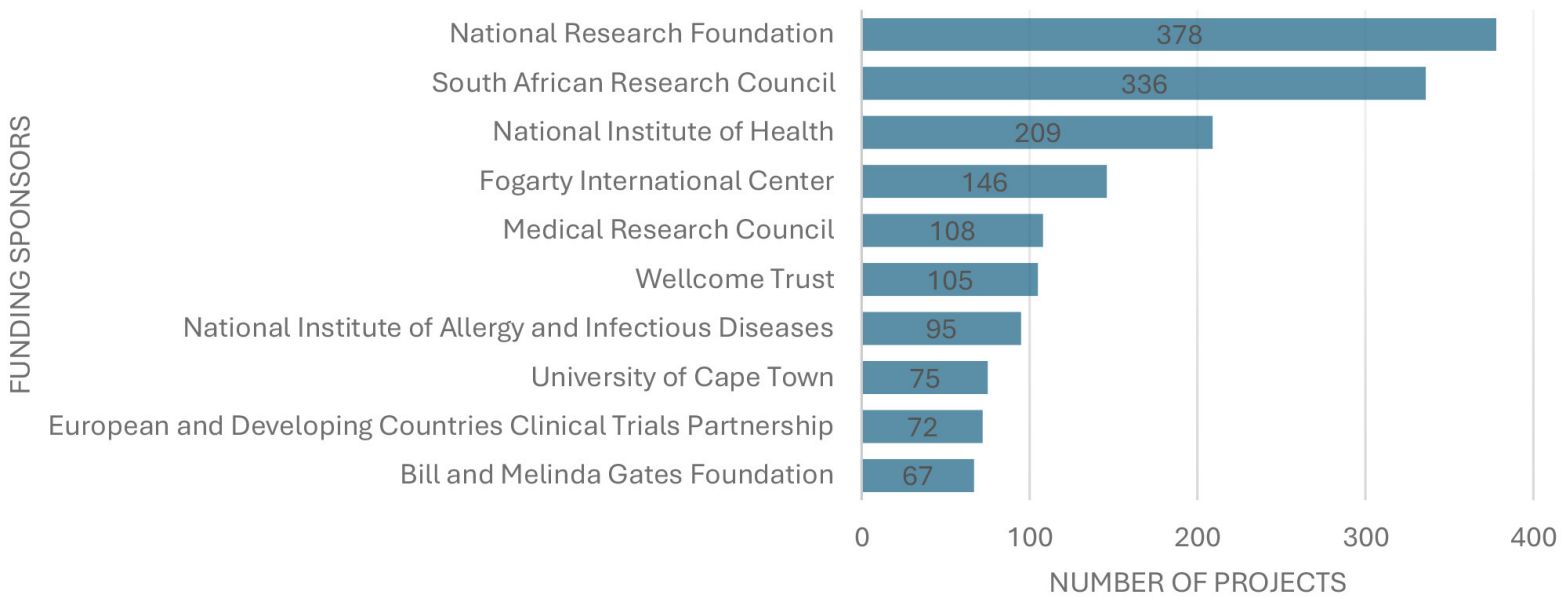
Ten topmost influential funding agencies.

According to the funding results, the National Research Foundation (NRF) and the South African Medical Research Council (SAMRC) have noticeably greater numbers of projects (Figure 3). In addition, other foreign funders from the USA and UK, such as the National Institute of Health (NIH), the Fogarty International Center (FIC) and the Medical Research Foundation and Wellcome Trust, are included in funding tuberculosis research in Africa.

According to the results of the analysis, the top cited documents on the topic of interest are Coovadia et al. (2009), with a total number of citations (TCs) of 876, followed by Boutayeb (2006), with 441 TCs. Interestingly, Boulle A (2021, Clin Infect Dis) has 332 TCs and the highest citation rate per year (83.00), which is an indication of recent impact or high relevance. Citations are often found in different scientific journals such as *Lancet, Clinical and Infectious Disease*, and *American Journal of Respiratory and Critical Care Medicine*. Additionally, papers are published in a variety of fields, including medicine, infectious diseases, and microbiology. With respect to more recent publications, citation rates are lower for older studies; for example, Diacon AH (2003) and Byarugaba DK (2004) report yearly citation rates of 13.68 and 13.43 TCs/year, respectively (Table 3).

**Table 3:** Most global cited scientific papers on tuberculosis.

Position	Paper	DOI	TCs	TC/year
1	Coovadia et al., 2009, Lancet	10.1016/S0140-6736(09)60951-X	876	54.75
2	Boutayeb, 2006, Transactions of the Royal Society Tropical Medicine Hygiene	10.1016/J.Trstmh.2005.07.021	441	23.21
3	Zar Hj, 2005, Lancet	10.1016/S0140-6736(05)17702-2	416	20.80
4	Mayosi Bm, 2005, Circulation	10.1161/CIRCULATIONAHA.105.543066	358	17.90
5	Bongani Mm, 2014, New Engl J Med	10.1056/Nejmsr1405012	333	30.27
6	Boulle A, 2021, Clin Infect Dis	10.1093/Cid/Ciaa1198	332	83.00
7	Marais Bj, 2006, Am J Respir Crit Care Med	10.1164/Rccm.200511-1809SO	316	16.63
8	Diacon Ah, 2003, Eur Respir J	10.1183/09031936.03.00017103a	301	13.68
9	Quaye Ik, 2008, Trans R Soc Trop Med Hyg	10.1016/J.Trstmh.2008.04.010	284	16.71
10	Cholo Mc, 2012, J Antimicrob Chemother	10.1093/Jac/Dkr444	282	21.69
11	Byarugaba Dk, 2004, Int J Antimicrob Agents	10.1016/J.Ijantimicag.2004.02.015	282	13.43
12	Joubert E, 2008, J Ethnopharmacol	10.1016/J.Jep.2008.06.014	274	16.12
13	Warren Rm, 2004, Am J Respir Crit Care Med	10.1164/Rccm.200305-714oc	252	12.00
14	Michel Al, 2010, Vet Microbiol	10.1016/J.Vetmic.2009.08.029	232	15.47
15	Ruwizhi N, 2020, Int J Mol Sci	10.3390/Ijms21165712	227	45.40
16	Wood R, 2007, Am J Respir Crit Care Med	10.1164/Rccm.200606-759 °C	222	12.33
17	Mcilleron H, 2006, Antimicrob Agents Chemother	10.1128/AAC.50.4.1170–1177.2006	220	11.58
18	Bedelu M, 2007, J Infect Dis	10.1086/521114	218	12.11
19	Ben-Ali M, 2004, Clin Diagn Lab Immunol	10.1128/CDLI.11.3.625-626.2004	208	9.90
20	Dookie N, 2018, J Antimicrob Chemother	10.1093/Jac/Dkx506	203	29.00
21	Zar Hj, 2007, Br Med J	10.1136/Bmj.39000.486400.55	202	11.22
22	Aderibigbe Ba, 2017, Molecules	10.3390/Molecules22081370	201	25.13
23	Anorlu Ri, 2008, Reprod Health Matters	10.1016/S0968-8080(08)32415-X	199	11.71
24	Louw Ge, 2009, Antimicrob Agents Chemother	10.1128/AAC.01577-08	198	12.38
25	Schnippel K, 2018, Lancet Respir Med	10.1016/S2213–2600(18)30235-2	196	28.00

**Figure 4:**
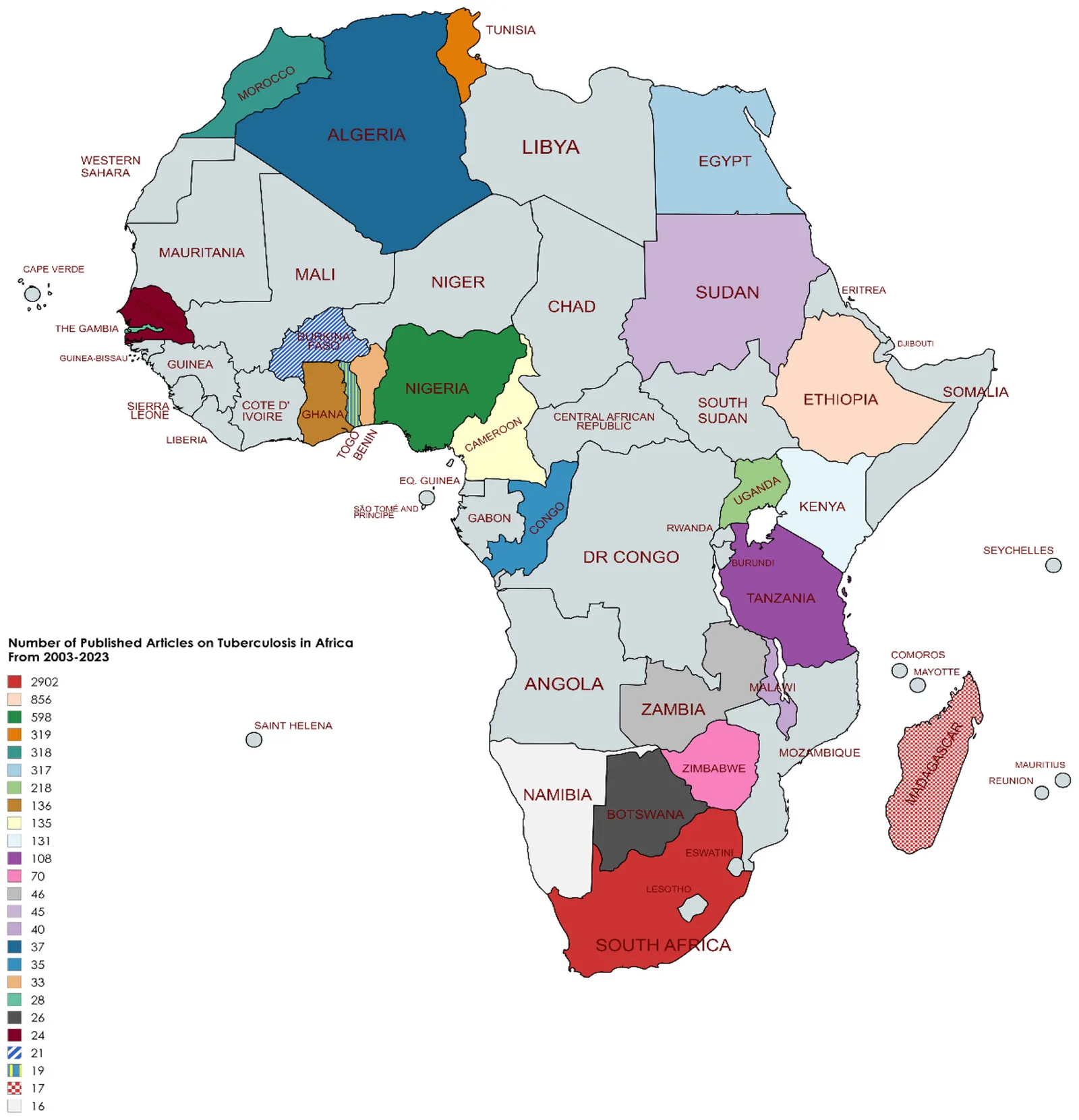
Published articles on tuberculosis in Africa between 2003 and 2023.

The results from the scientific documents retrieved from the Scopus database on tuberculosis from 2003–2023 revealed that South Africa has the highest total number of articles (*n* = 2,902), followed by Ethiopia (*n* = 856), Nigeria (*n* = 598), Tunisia (*n* = 319), and Morocco (*n* = 318; Figure 4).

Table 4 displays the top 20 African countries that have been cited most frequently from the documents retrieved from the Scopus database on tuberculosis from 2003–2023, using the institution or university affiliation of at least one author in each published paper to estimate the most cited countries. South Africa has the highest total number of citations (55.425) and the highest average number of citations per article (19.10). Ethiopia and Nigeria have lower citation averages (12.40 and 8.70, respectively) but a significant number of article citations. Gambia stands out for an exceptionally high average number of citations per article (41.60), despite having a relatively low total number of article citations (1.164), whereas Zimbabwe, Zambia, Malawi, and Namibia also have notable average citation rates, ranging from 12.90–13.90. However, Tunisia, Morocco, and Cameroon have lower average citation rates than others do but have a considerable number of article citations. Finally, Botswana, Benin, Sudan, and Congo have fewer article citations and relatively modest citation averages, ranging from 9.10–13.90 (Table 4).

**Table 4:** The 20 African countries most cited in tuberculosis research from 2003–2023.

Position	Country	Total citation	Average article citations
1	South Africa	55,425	19.10
2	Ethiopia	10,648	12.40
3	Nigeria	5,205	8.70
4	Egypt	3,813	12.00
5	Uganda	2,515	11.50
6	Tunisia	2,026	6.40
7	Morocco	1,994	6.30
8	Cameroon	1,938	14.40
9	Kenya	1,672	12.80
10	Ghana	1,535	11.30
11	Tanzania	1,241	11.50
12	Gambia	1,164	41.60
13	Zimbabwe	904	12.90
14	Zambia	661	14.40
15	Malawi	538	13.40
16	Benin	420	12.70
17	Sudan	410	9.10
18	Congo	320	9.10
19	Botswana	244	9.40
20	Namibia	222	13.90

Overall, while South Africa leads in both metrics, Gambia has the highest average citation rate, and other countries have relatively high average citations despite having fewer total articles.

### Most relevant authors on tuberculosis publications in Africa

**Figure 5:**
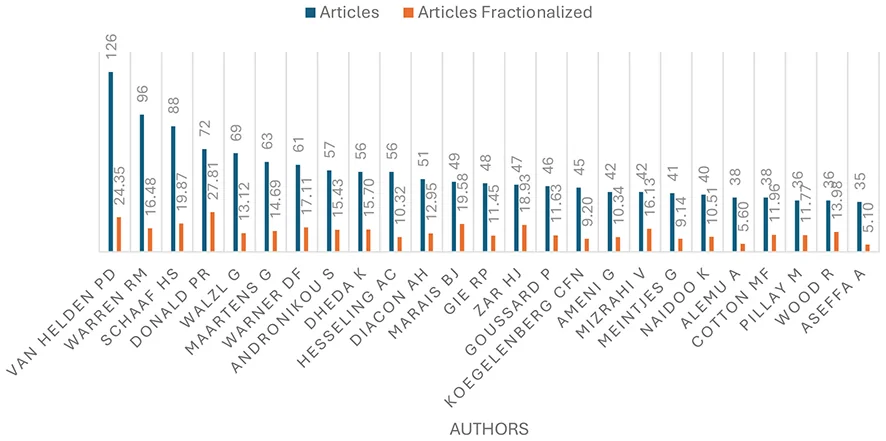
Shows the 25 top authors with the most publications related to TB from the Scopus database from 2003–2023.

Figure 5 shows the 25 topmost relevant authors on TB published papers recovered from the Scopus database from 2003–2023. The results show the authors with the highest total publication counts with their fractionalized article counts, indicating their relative contribution to the field. Van Helden PD has the highest number of published articles (*n* = 126), followed by Warren RM, Schaaf HS, Donald PR, Walzl G, and Maartens G, with 96, 88, 72, 69, and 63 articles, respectively (Figure 5). Donald PR has the highest fractionalized article count of 27.81, indicating a high level of contribution when considering fractional authorship. Additionally, Van Helden PD has a high fractionalized count of 24.35, indicating a significant contribution relative to the total number of articles published. Other notable authors include Maartens G, Warner DF, and Marais BJ, with substantial fractionalized counts of 14.69, 17.11, and 19.58, respectively, thus reflecting their significant impact. Analysis of leading authors identifies key contributors and collaboration hubs that influence research capacity development in Africa.

In general, the fractionalized count offers information about the authors' respective effects and collaborative contributions, whereas the total number of publications is a measure of production.

### Analysis of the most productive institutions and their collaboration network

**Figure 6:**
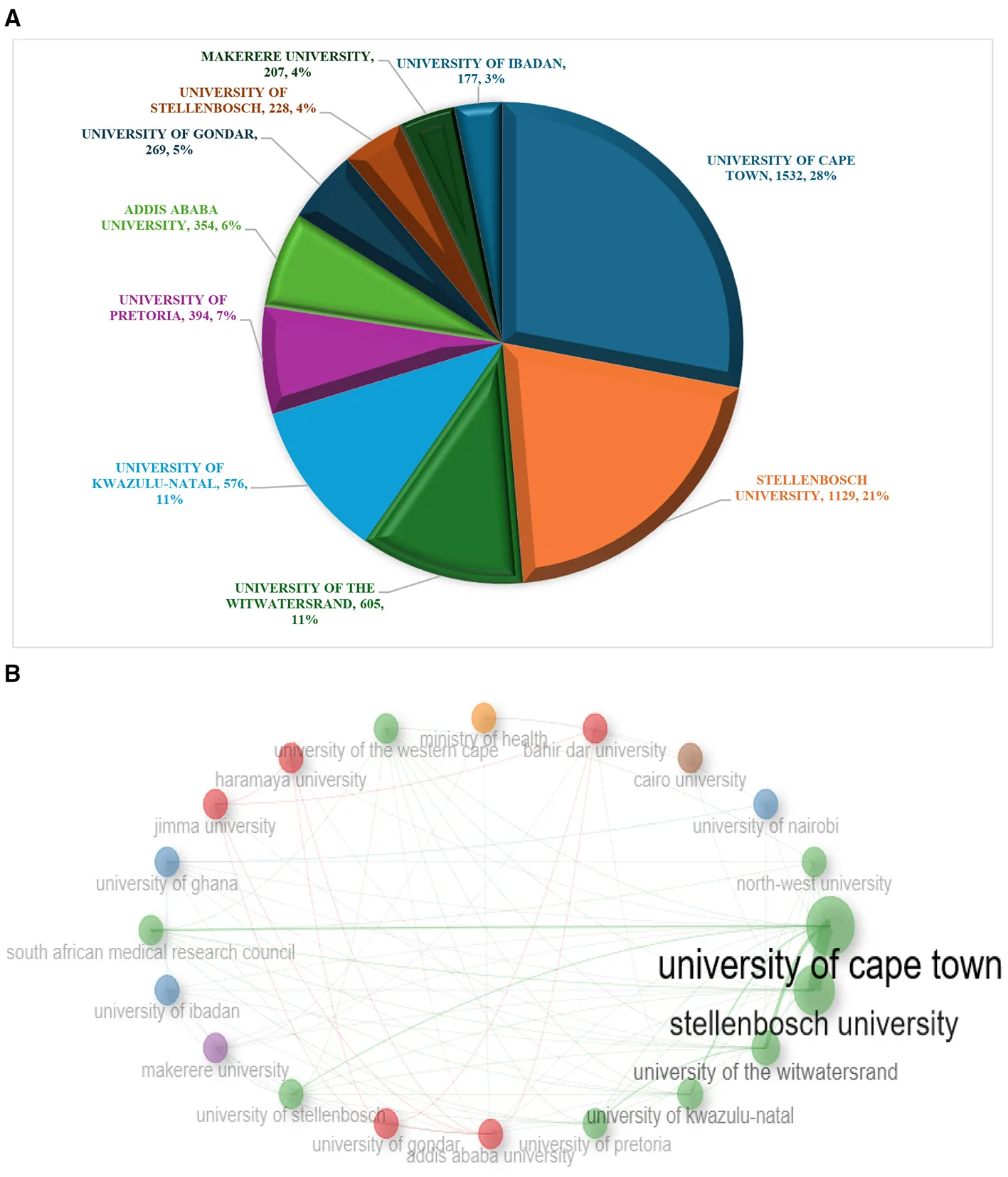
**(A)** The most productive institutions on tuberculosis in Africa. **(B)** Collaboration network among institutions on tuberculosis research in Africa.

The results of this analysis reveal the most prolific institution in terms of research output on tuberculosis on the African continent. The University of Cape Town (*n* = 1.532; 28%) is the leading and most prolific institution in terms of the number of publication outputs from 2003–2023, followed by Stellenbosch University, University of Witwatersrand, and University of KwaZulu-Natal, with publication outputs of 1.129 (21%), 605 (11%), and 576 (11%), respectively (Figure 6A). In addition, the University of Pretoria and Addis Ababa University contributed significantly to the number of article publications, with 394 (7%) and 354 (6%), respectively.

Compared with other institutions, the University of Cape Town and Stellenbosch University generally have strong research outputs. The top five collaborative networks among institutions were determined to be between (i) the University of Cape Town, (ii) Stellenbosch University, (iii) the University of Witwatersrand, (iv) the University of KwaZulu-Natal, and (v) the University of Pretoria (Figure 6B). This was determined based on the leading eigenvalue clustering algorithm that assesses betweenness centrality.

**Figure 7:**
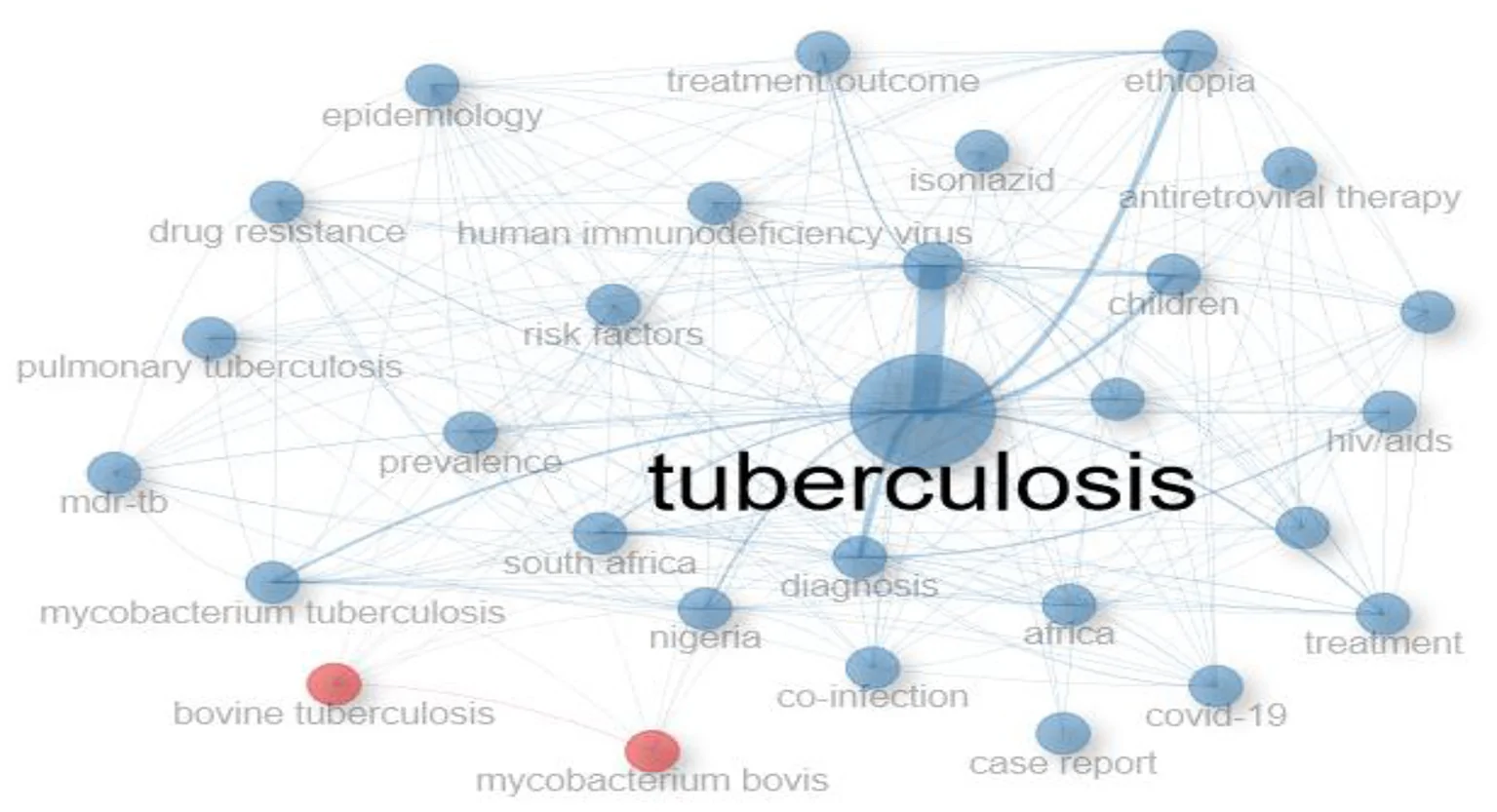
Title word co-occurrence network in published papers on tuberculosis in Africa.

We further determined the title word co-occurrence by determining the number of occurrences, correlations, and co-occurrences of each title word in published papers on tuberculosis between 2003 and 2023 via the Walktrap clustering algorithm, which assesses betweenness centrality. Figure 7 displays the title word co-occurrence network analysis, correlations, and nodes of the 30 most used terms in published papers on tuberculosis on the African continent. The width or size of each node indicates the frequency of occurrence of each title word in published papers on tuberculosis research on the African continent.

**Figure 8:**
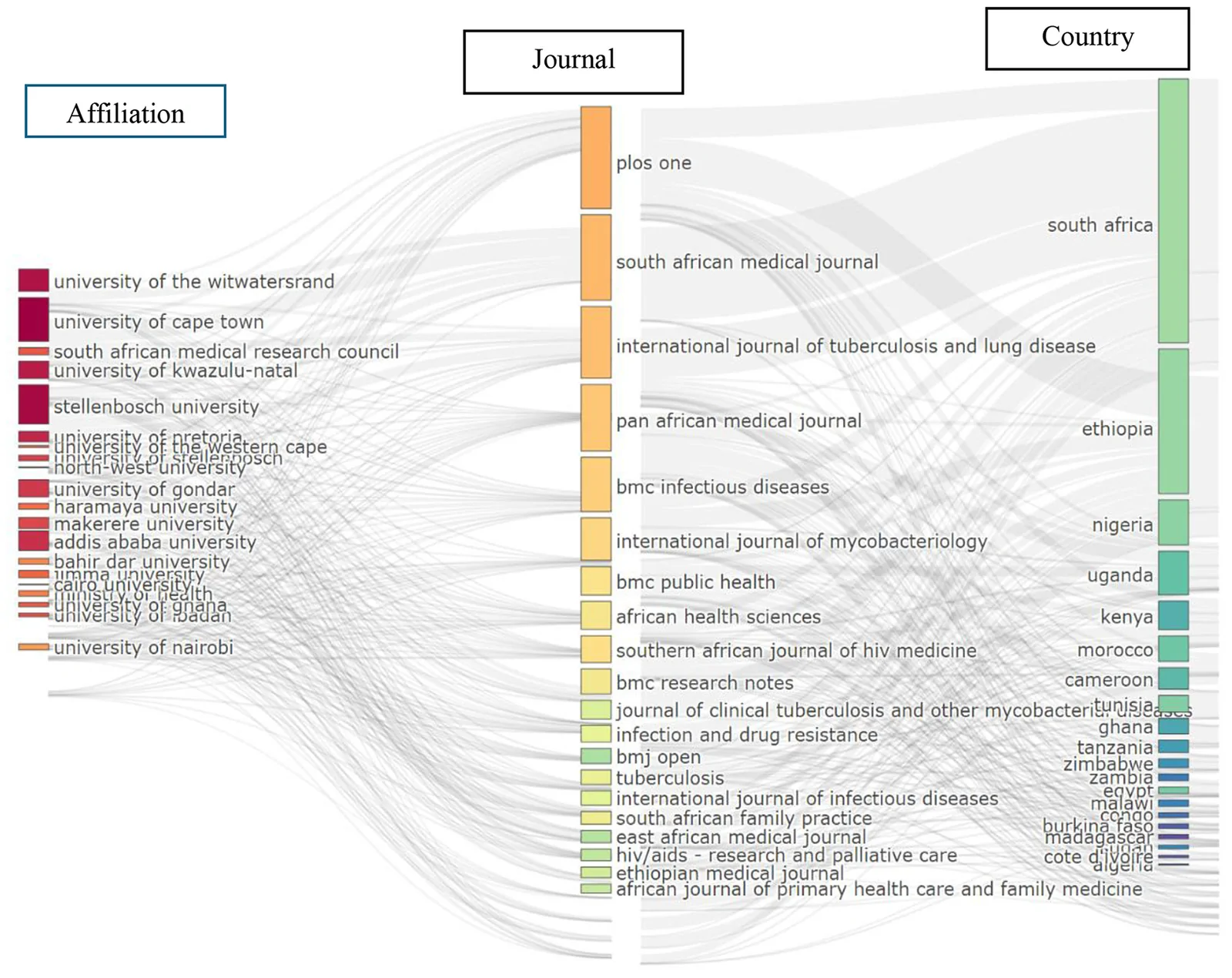
Three-field plot: correlations between Affiliation **(left)**, Journal **(middle)**, and Country **(right)**.

Three-field plot showing the inflow and outflow of the top 10 affiliations, journals, and countries that have contributed to tuberculosis research on the African continent from 2003−2023 (Figure 8). Our three-field graphic, in which the height of the rectangular nodes in the collaboration network is correlated with the frequency of occurrence, shows the incoming and outgoing flows between these fields.

**Figure 9:**
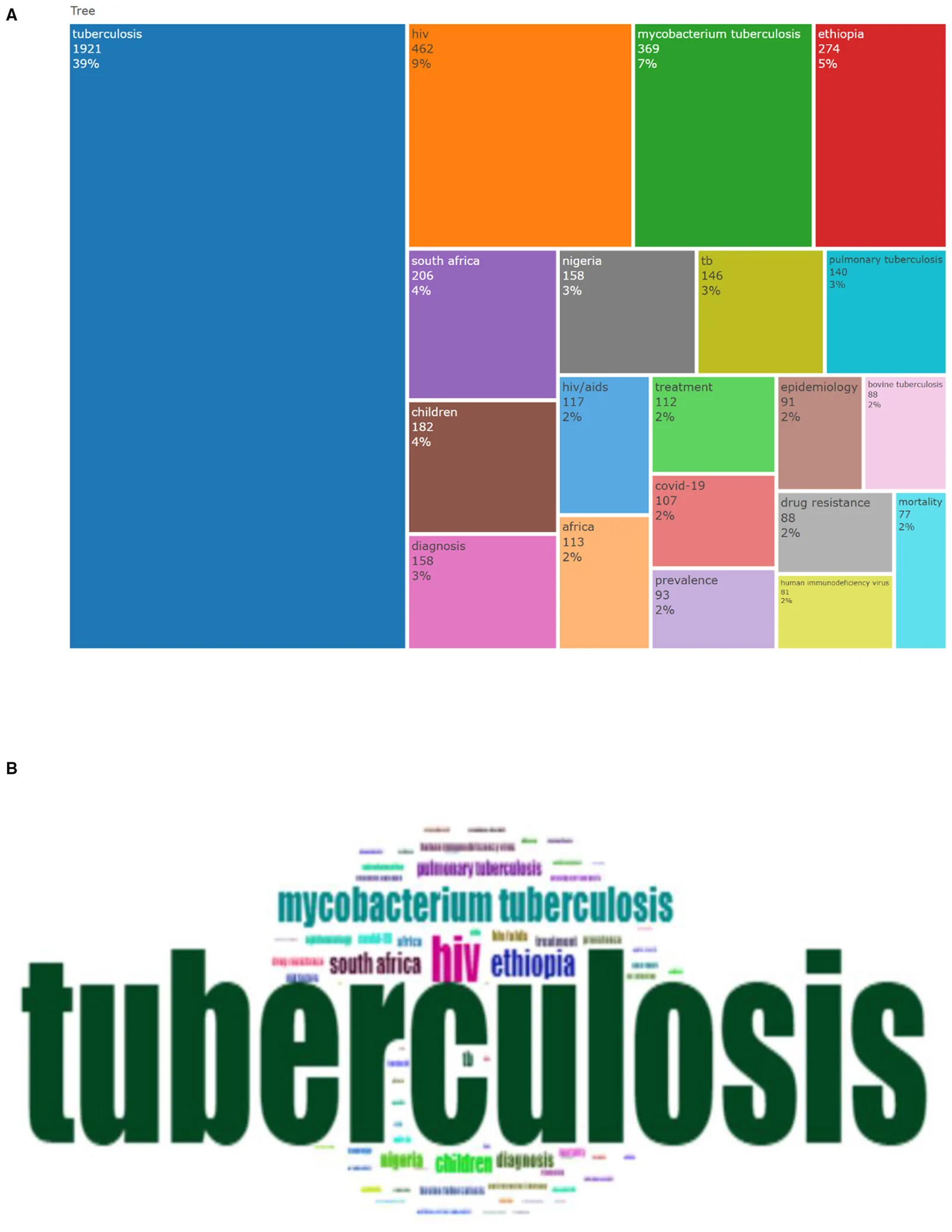
**(A)** TreeMap depicting the top 20 authors' keywords and **(B)** WorldCloud using “keywords plus” used in research on tuberculosis in Africa from 2003–2023.

The displayed TreeMap shows the top 20 keywords plus in tuberculosis research outputs between 2003 and 2023. A larger rectangular area represents a larger proportion of a particular term. The top three keywords used by the authors were tuberculosis (39%), HIV (9%), and *Mycobacterium tuberculosis* (7%; Figures 9A, B). Additionally, we generated a word cloud via the “Biblioshiny App” from Bibliometrix in R Studio. We selected “keywords plus” in the graphical parameters, which was motivated by its effectiveness in reflecting the content of articles and tends to be more broadly descriptive, as well as being able to capture a wider range of scientific concepts than “authors keywords” (Zhang et al., 2016). A maximum of 50 words was selected, and random dark and circular symbols were selected for the text colors and shapes of the figures, respectively, while the padding values and ellipticity were 2 and 1.0, respectively.

**Figure 10:**
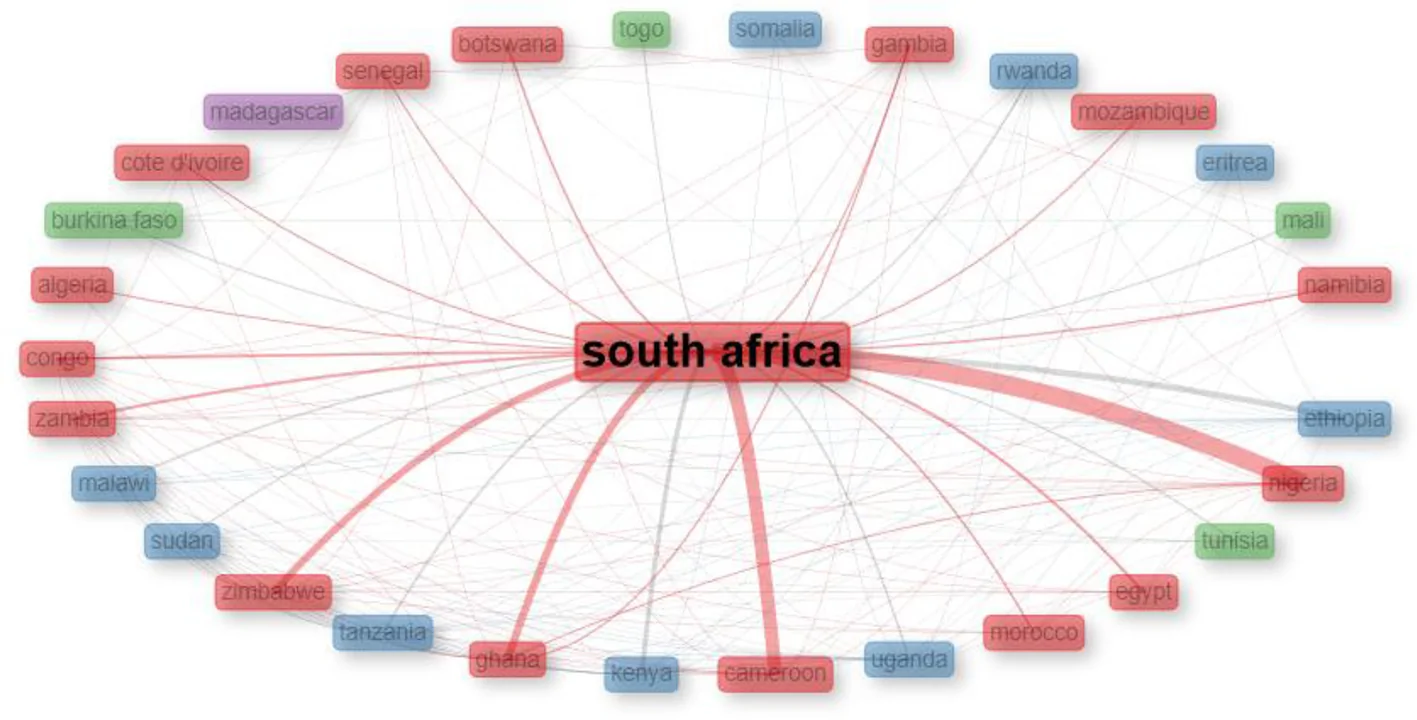
Country's collaboration network.

In relation to collaboration networks among countries, Figure 9 shows the visualization analysis of the collaboration among researchers working on TB in published papers in African countries from 2003–2023. The diameter or size of each node indicates the number of times the author's country collaborates with other African countries, while the lines display the networks that facilitate collaboration between each country, and each node represents a distinct African country. The clusters show that South Africa has the highest number of collaboration networks, followed by Nigeria, Cameroon, Ghana, and Zimbabwe (Figure 10). The degree of collaboration between two countries is indicated by the thickness of their respective matrices; for example, the highest degree of collaboration network or strength is demonstrated by the matrices between South Africa and Nigeria.

**Figure 11:**
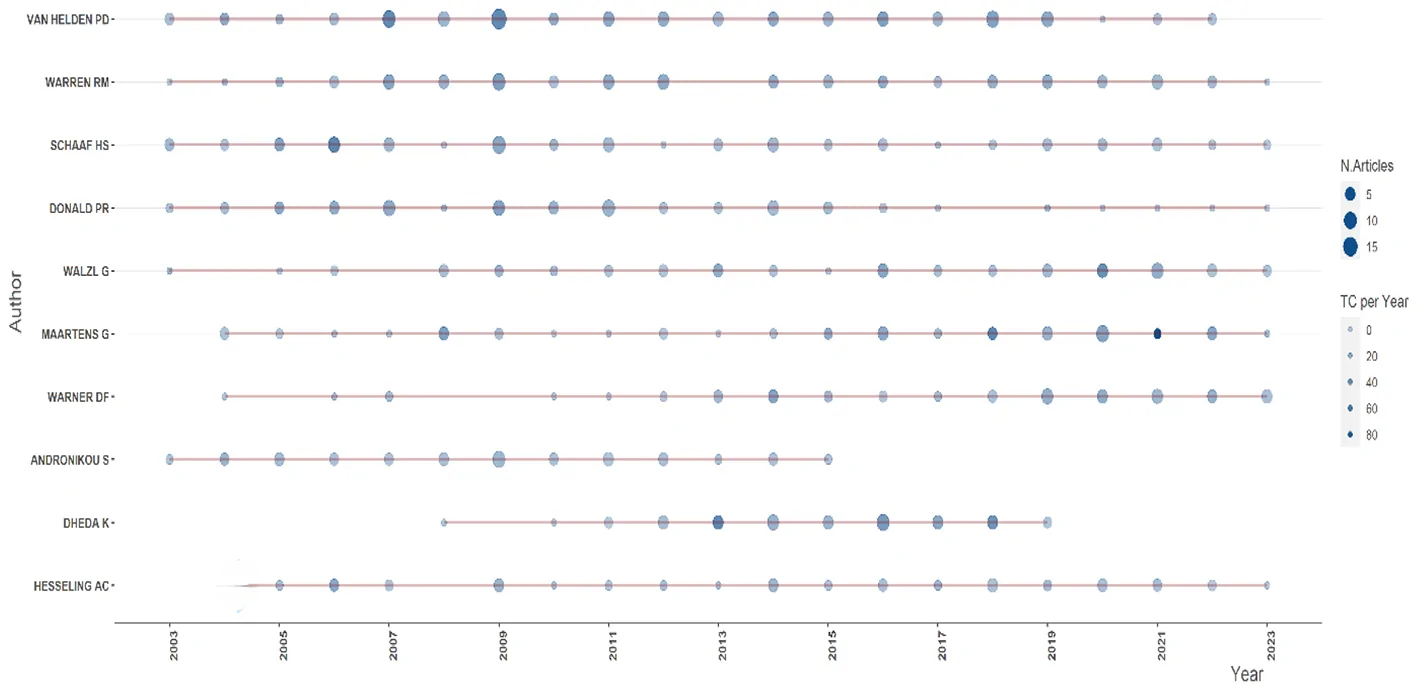
Author's production over time.

Display the top 10 most prolific authors during the study period. The total number of citations per year is represented by the strength of the colors, and the size of the dots indicates the number of publications. Among the top 10 prolific authors, Van Helden PD has the highest number of published papers (*n* = 126), followed by Warren RM (*n* = 96) and Schaaf HS (*n* = 88). In terms of the frequency of publications during the study period, Van Helden PD (highest frequency of 18 publications in 2009) and Schaaf HS (highest frequency of 12 publications in 2009) display the highest peak in annual publication frequency, indicating significant research output during certain years (Figure 11).

In addition, Andronikou S, publications ranged from 2–10 per year, with the highest frequency of 10 publications in 2009, with notable spikes in 2005, 2008, and 2011, whereas Dheda K, has publications ranging from 1–10 per year, with the highest frequency of 10 publications in 2016, with a notable peak (*n* = 8) in 2014.

A thematic analysis of the documents was conducted, and the authors' keywords were used to identify the conceptual and functional structure of the study. This helps to evaluate the key research themes, textual knowledge and conglomerate classes of the research area. The matic map divides the research themes into two factors, i.e., density (X-axis) and centrality (Y-axis) (Nasir et al., 2020). The X-axis represents the importance of a study theme and shows the network cluster or the degree of interaction with other graph clusters. Density measures the internal strength and theme growth of a cluster network.

**Figure 12:**
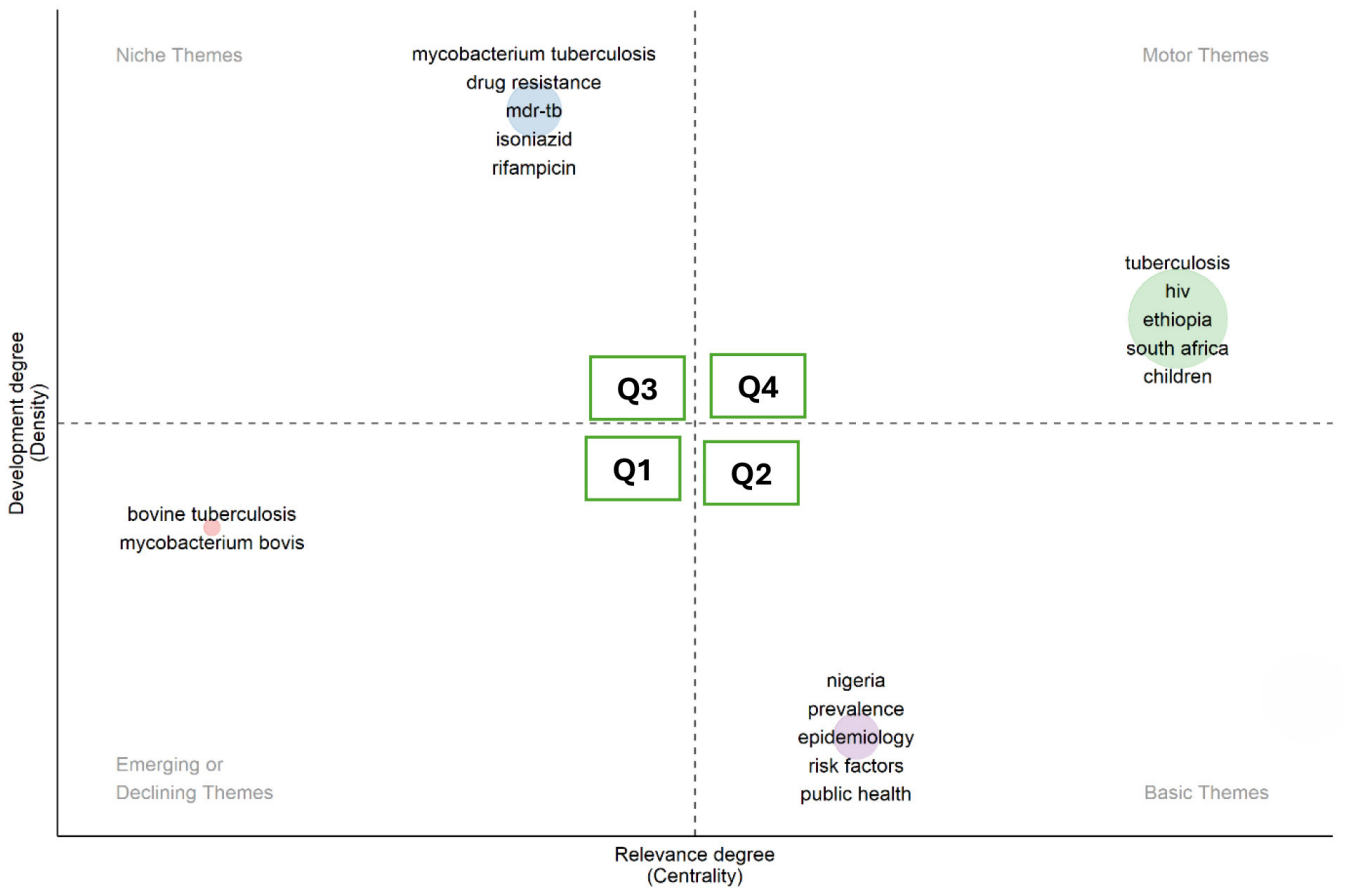
Visualization of thematic mapping.

As shown in Figure 12, there are four distinct theme types according to their degree of centrality and density: the motor theme (upper right), the niche theme (upper left), the basic theme (bottom right), and the emerging or declining theme (lower left) (Delecroix and Epstein, 2004).

In this study, Q1 (emerging or declining themes) denotes a theme that has not yet developed fully and has low centrality and density. A network will gradually develop topics such as bovine tuberculosis and *Mycobacterium bovis*. These subjects are of less importance but have potential for future research. Q2 (basic themes) indicates that basic, transversal, and underlying themes have relevance probabilities with minimal network development. In the future, greater attention must be paid to topics such as prevalence, epidemiology, public health, and risk factors. Q3 (niche themes) indicates well-developed themes. Potential topic areas such as tuberculosis, diagnosis, and treatment should receive more attention. Q4 (motor themes) denotes well-developed and central themes. Keywords such as prevalence, epidemiology, and pulmonary tuberculosis are highly important and helpful for advancing tuberculosis research in Africa.

## Discussion

Recently, bibliometrics has become an essential tool and has garnered significant attention within the scientific community owing to its practical approach to assessing the merits, advancements, and research trends in a particular field via several bibliometric indices (Sugimoto et al., 2019; Alvarez-Betancourt and Garcia-Silvente, 2014). It has also become a tool that specifically addresses the broad overlap and fusion of information science, statistics, mathematics, and philology (He et al., 2017). This tool also offers a novel concept and a wide range of applications in different study domains, making it an essential research platform; hence, it is utilized in different fields of study, including medical and health sciences (Lou et al., 2020; Islam et al., 2022), business and economics (Merigó et al., 2016), and sustainable energy (Hache and Palle, 2019).

For this bibliometric study, a total of 8,181 documents were retrieved from the Scopus database after applying filters and different parameters on the search engine for different analyses to understand the trends and gaps in published scientific documents on TB research on the African continent from 2003–2023. Within the period of study, we noticed convincing evidence of a continuous upwards trend in the number of publications on research on tuberculosis in Africa, with the lowest (*n* = 105) number of publications occurring in the year 2003 and the highest (756) number of publications occurring in the year 2021, which is similar to the findings of another 10-year bibliometric analysis on TB and TB-related research trends in Africa (Igwaran and Edoamodu, 2021). However, the observed increase in publication output should not be interpreted solely as evidence of increased research capacity. Publication growth may also reflect broader journal coverage within Scopus, increased international research collaboration, improvements in indexing practices, and greater visibility of African research over time. Therefore, publication counts should be interpreted as indicators of research activity rather than direct measures of national research capacity. Recent decreases in citation counts or publication numbers should be interpreted cautiously because newly published articles require time to be indexed and cited in subsequent literature (Figure 1A). Temporary declines in publication output around 2020–2022 may be associated with COVID-19 disruptions to laboratory work and clinical trials, which have been documented in global health research during the pandemic (Margas et al., 2022). The COVID-19 pandemic also appears to have influenced tuberculosis research trends during the latter part of the study period. Disruptions to tuberculosis diagnostic services, treatment programmes, laboratory activities, and clinical research shifted research priorities toward maintaining essential tuberculosis services during the pandemic, while also increasing interest in the interaction between COVID-19 and tuberculosis. Most published articles about interest were published in high- and low-impact factor journals, including the *International Journal of Infectious Diseases* (IF = 8.4), *International Journal of Tuberculosis and Lung* (IF = 4.0), *BMC Infectious Diseases* (IF = 3.7), and *BMC Public Health* (IF = 4.5; Table 2).

The impact factor of a journal and the total number of document or journal citations are two key factors used to determine a paper's scientific or academic influence; the citation index is solely recognized as a measure of identifiability (Tsai et al., 2006; Sobhy, 2016). Although citation-based indicators provide useful measures of scientific influence and visibility, they should not be interpreted as direct indicators of research quality (Tsai et al., 2006). Citation counts are affected by several factors, which includes publication age, journal visibility, collaboration networks, research topic, and self-citation practices. Thus, highly cited publications reflect greater scholarly attention rather than necessarily representing higher scientific quality. Similarly, the higher citation rates observed for some publications may partly reflect their longer availability within the scientific literature, publication in high-visibility journals, and participation in international collaborative networks, all of which can substantially influence citation accumulation over time. Journal impact factor reflects average citation performance of journals rather than the influence of individual articles. Several African journals without impact factors still play an important role in disseminating regionally relevant tuberculosis research. In addition, the most cited published article retrieved from Scopus on tuberculosis was published in *Lancet*, with a total citation of 876 (Coovadia et al., 2009), followed by *Transactions of the Royal Society Tropical Medicine Hygiene*, with a total citation of 441 (Boutayeb, 2006). Furthermore, South Africa was among the topmost cited countries (*n* = 55,425), followed by Ethiopia (*n* = 10,648) and Nigeria (*n* = 5,205). These comparisons should be interpreted cautiously because absolute publication and citation count naturally favor countries with larger research systems, stronger research infrastructure, and greater funding investment. Although normalized indicators such as publications per capita, field-weighted citation impact, or citations per publication would provide additional context, such metrics were beyond the scope of the present bibliometric analysis. Consequently, our comparisons primarily reflect relative research output and scholarly visibility rather than overall national research performance. A measure of the quality of the document is the total number of citations to the top-cited publications in this study. The quantity of accumulated documents or articles can differ across different search engines, which is why citation counts can fluctuate (Shao and Zheng, 2016). We also analyzed the topmost productive institutions and their collaboration network and discovered that the five topmost productive institutions were from South Africa: (i) the University of Cape Town with 1,532 totals; (ii) Stellenbosch University; (iii) the University of Witwatersrand; (iv) the University of KwaZulu-Natal; and (v) the University of Pretoria. Compared with other institutions, the University of Cape town and Stellenbosch University produced strong research outputs. However, higher publication output should not be interpreted as a direct measure of institutional research excellence. Institutional productivity is influenced by factors such as funding availability, research workforce size, long-established research programmes, international collaborations, and institutional policies that support scientific publishing. In this study, the number of published papers on tuberculosis among different authors from different universities or institutions was used to determine the most productive institutions and their collaboration network. The lines indicate collaboration networks between institutions or universities, and the thickness of each line indicates the degree or strength of collaboration.

Most of the published papers on tuberculosis in Africa, according to our data, were from South Africa, which is not surprising considering that South Africa is one of the countries with the highest incidence of TB (WHO, 2023; Conan et al., 2022), especially with HIV/TB coinfection, which has exerted immense pressure on the healthcare system in Africa. Conspicuously, the frequency of HIV/TB coinfection is highest in Africa, with over 50% of cases occurring in southern Africa (Olivier and Luies, 2023). Different funding agencies invest more in TB research in South Africa than in other African countries do (Figure 2). The pre-dominance of major national and international funding agencies suggests that sustained financial investment has been an important driver of tuberculosis research productivity in Africa. Funding not only supports individual research projects but also facilitates multicentre collaborations, advanced laboratory infrastructure, post-graduate training, and participation in international clinical trials, all of which contribute to increased publication output and research visibility. The strong institutional collaboration observed among leading universities is therefore likely to be influenced, at least in part, by these long-term funding investments. Given that South Africa is one of six countries in the world that accounts for 60% of the global tuberculosis burden, the high rate of institution production outputs and their network collaboration focusing on tuberculosis on the African continent may also be a result of the high prevalence of tuberculosis in the country. Additionally, the high incidence rate of HIV/TB coinfection in South Africa could be another factor for increased interest in conducting and investing heavily in TB research, innovative treatment, and clinical trials (Shamu et al., 2019; Yaghoubi et al., 2020; Weinrib et al., 2020). The prominence of HIV/TB co-infection as one of the dominant research themes reflects the unique epidemiological context of sub-Saharan Africa, where HIV infection substantially increases susceptibility to active tuberculosis. The overlapping epidemics have driven extensive research on diagnosis, treatment outcomes, prevention strategies, and integrated HIV/TB healthcare delivery, making HIV/TB co-infection a sustained priority over the past two decades. Similarly, multidrug-resistant tuberculosis emerged as a major research priority due to increasing resistance to first-line anti-tuberculosis drugs, growing treatment complexity, prolonged treatment duration, and the associated public health and economic burden. These challenges stimulated considerable investment in molecular diagnostics, surveillance programmes, and therapeutic research throughout the study period. We further observed that Addis Ababa University was one of the top 10 institutions with a high volume of article production outputs (Figure 5A), possibly because of the high prevalence of TB, MDR-TB, and TB-HIV coinfection in Ethiopia. Ethiopia remains among the countries with high TB burdens worldwide, with an annual TB incidence of 119 cases per 100,000 people (Meaza et al., 2023). Our results on the institution collaboration network for TBs showed that the University of Cape Town has the highest degree of collaboration, followed by Stellenbosch University. Compared with other local and foreign institutions, these universities also showed the strongest collaborations (Figure 5B), which is a measure of the closeness centrality between the nodes, showing direct influence between the institutions (Wenwen et al., 2019). A collaborative research network can help other researchers expand their field of research or join other scientific research groups in conducting allied studies, which will accelerate the generation of new ideas and resources, resulting in higher-quality research outcomes, which may not be possible if they work alone (Wenwen et al., 2019; Duan, 2024). The prominence of collaborative networks observed in this study may also contribute to higher publication output and citation visibility because internationally collaborative studies frequently benefit from broader dissemination, larger multidisciplinary teams, and wider readership. Academic research today is characterized by scientific network collaboration and is believed to be advantageous for integrating different skill sets to address practical issues in the quest to provide answers to challenges in science, economy, and society that frequently call for or demand interdisciplinary methods (Thelwall and Maflahi, 2020). Our study also analyzed the title word co-occurrence network in published documents on tuberculosis from 2003–2023 via author keywords. By offering insights into the major themes and subjects discussed in a publication, analyzing the co-occurrence network contributes significantly to bibliometric analysis and helps researchers better understand the focus of their work, discover connections and relationships between various concepts and themes within a field of study, and locate pertinent studies (Liu and Prajapati, 2022; Vătămănescu and Vintilă, 2023). Researchers are able to determine research hotspots, trends, and the frequency of terms over time by analyzing word co-occurrence networks (Vătămănescu and Vintilă, 2023). The three-field plot analysis in our study presented a visualization of the interrelationship between affiliations, journals, and countries (Figure 7) to understand the incoming and outgoing flows among the top 20 countries that have contributed significantly to tuberculosis research in Africa from 2003–2023. The results revealed that South Africa has a greater number of university affiliations than other African countries in terms of tuberculosis research outputs between 2003 and 2023. To display the top 20 keywords from the included published papers on tuberculosis in Africa from 2003–2023, a tree map was created. We further examined the most frequent research keywords (Figure 8B). We discovered that there was a positive correlation between font size and word frequency, meaning that the words that were used more frequently in the word cloud had larger font sizes. We further analyzed the author's country collaboration network and discovered that South Africa has the highest number of collaboration networks, followed by Nigeria, Cameroon, Ghana, and Zimbabwe. This is slightly different from what (Igwaran and Edoamodu, 2021) projected in their bibliometric study. This could be a result of the different databases used and the different periods of study. Finally, we explored author production from 2003–2023 and discovered that among the top 10 prolific authors, Van Helden PD has the highest number of published papers (*n* = 126), followed by Warren RM (*n* = 96) and Schaaf HS (*n* = 88). In terms of the frequency of publications during the study period, Van Helden PD (highest frequency of 18 publications in 2009) and Schaaf HS (highest frequency of 12 publications in 2009) display the highest peak in annual publication frequency, indicating significant research output during certain years.

Finally, a thematic map was created to understand the status of tuberculosis research on the African continent and analyse the future direction of research development within the field (García-Lillo et al., 2023). The continued importance of bovine tuberculosis is evident from its substantial implications for animal health, public health, food security, and biodiversity conservation. Caused by *Mycobacterium bovis, bovine tuberculosis* remains a significant zoonotic disease affecting cattle, wildlife, and humans, particularly in low- and middle-income countries where livestock production is closely linked to rural livelihoods (Kock et al., 2021). In Africa, transmission between livestock and wildlife reservoirs, particularly African buffalo, together with the potential role of warthogs and other wildlife species in disease persistence, underscores the complexity of controlling bovine tuberculosis within a One Health framework (Roos, 2022; Arnot and Michel, 2020). These interconnected transmission pathways highlight the need for integrated surveillance systems, improved diagnostic approaches, and collaborative disease control strategies across the human, livestock, and wildlife sectors (Arnot and Michel, 2020). Furthermore, recent evidence suggests that warthogs may serve as valuable sentinel species for monitoring disease transmission in endemic ecosystems, providing additional opportunities to strengthen surveillance and early detection programmes (Roos, 2022). From a policy perspective, the thematic map provides an evidence-based approach for identifying strategically important but comparatively underdeveloped research areas that may benefit from increased investment (Radosveta et al., 2025). Policymakers and funding agencies can use these findings to prioritize research on bovine tuberculosis through dedicated funding for surveillance, molecular epidemiology, wildlife–livestock interface studies, diagnostic innovation, and One Health implementation programmes. Such investments are timely given by the World Health Organization's continued emphasis on sustained research investment and strengthened surveillance to achieve the End TB Strategy, while recognizing that funding gaps continue to threaten global tuberculosis control efforts (WHO, 2025). Similarly, long-standing bovine tuberculosis eradication programmes demonstrate that sustained surveillance, coordinated animal health policies, and continued investment remain essential for reducing disease transmission and protecting both animal and public health (U. S. Department of Agriculture, 2026). Collectively, these findings suggest that strengthening research on bovine tuberculosis could contribute substantially to integrated tuberculosis control strategies across Africa while addressing important knowledge gaps at the human-animal-environment interface.

As one of the first studies to explore the trends and advancements of tuberculosis research outputs on the African continent between 2003 and 2023, using a widely accepted and valued scientific platform, we conducted a thorough analysis, displayed, and analyzed the metadata in a comprehensible manner. However, it is imperative to highlight the limitations of our bibliometric study. We used only one scientific database (Scopus) for our search, and we filtered it into only documents published in the English language to increase the reliability of the results. To provide a comprehensive study about interest in African countries, we included book chapters, conference papers, early access publications, and review articles, which might have resulted in different results that focused only on journal articles. Although the Scopus database served as the foundation for this study's conclusions, we acknowledge that the results from other databases, including Web of Science (WoS), might not be statistically significant. Therefore, it is highly recommended that future research conducts in-depth analyses on tuberculosis via multiple databases, such as WoS, PubMed, Dimension, and Google Scholar. Since no database is perfect and certain inclusion criteria may be biased by overrepresenting journals that utilize the English language, bibliometric results should always be evaluated cautiously.

## Limitations

This bibliometric analysis has several limitations. First, the search strategy included only publications with at least one author affiliated with an African institution. Although this approach was appropriate for evaluating African research capacity, collaboration, and institutional productivity, it may have excluded studies conducted on African populations by research teams whose authors were affiliated exclusively with institutions outside Africa. Consequently, the findings should be interpreted as representing tuberculosis research with documented African institutional participation rather than the complete global body of tuberculosis research relating to Africa. Future bibliometric studies could combine affiliation- and topic-based approaches to provide a more comprehensive assessment of tuberculosis research concerning African populations.

## Data Availability

The data and analytical materials supporting the findings of this study have been deposited in the Zenodo repository and are publicly available at https://doi.org/10.5281/zenodo.21672964. The repository contains the search strategy, processed bibliometric datasets, analytical workflow, and supplementary materials used in this study. Raw Scopus records are subject to Elsevier licensing conditions and therefore only materials that can be legally shared are included in the repository.
